# Assessment of population exposure to PM10 for respiratory disease in Lanzhou (China) and its health-related economic costs based on GIS

**DOI:** 10.1186/1471-2458-13-891

**Published:** 2013-09-27

**Authors:** Zhaobin Sun, Xingqin An, Yan Tao, Qing Hou

**Affiliations:** 1 Beijing Meteorological Observatory, Beijing 100089, China; 2 Chinese Academy of Meteorological Sciences, China Meteorological Administration, Beijing 100081, China; 3 College of Atmospheric Sciences, Lanzhou University, Lanzhou 730000, China

**Keywords:** PM_10_, Population exposure assessment, Economic assessment, Generalized additive model (GAM), GIS method

## Abstract

**Background:**

Evaluation of the adverse health effects of PM_10_ pollution (particulate matter less than 10 microns in diameter) is very important for protecting human health and establishing pollution control policy. Population exposure estimation is the first step in formulating exposure data for quantitative assessment of harmful PM_10_ pollution.

**Methods:**

In this paper, we estimate PM_10_ concentration using a spatial interpolation method on a grid with a spatial resolution 0.01° × 0.01°. PM_10_ concentration data from monitoring stations are spatially interpolated, based on accurate population data in 2000 using a geographic information system. Then, an interpolated population layer is overlaid with an interpolated PM_10_ concentration layer, and population exposure levels are calculated. Combined with the exposure-response function between PM_10_ and health endpoints, economic costs of the adverse health effects of PM_10_ pollution are analyzed.

**Results:**

The results indicate that the population in Lanzhou urban areas is distributed in a narrow and long belt, and there are relatively large population spatial gradients in the XiGu, ChengGuan and QiLiHe districts. We select threshold concentration C_0_ at: 0 μg m^-3^ (no harmful health effects), 20 μg m^-3^ (recommended by the World Health Organization), and 50 μg m^-3^ (national first class standard in China) to calculate excess morbidity cases. For these three scenarios, proportions of the economic cost of PM_10_ pollution-related adverse health effects relative to GDP are 0.206%, 0.194% and 0.175%, respectively. The impact of meteorological factors on PM_10_ concentrations in 2000 is also analyzed. Sandstorm weather in spring, inversion layers in winter, and precipitation in summer are important factors associated with change in PM_10_ concentration.

**Conclusions:**

The population distribution by exposure level shows that the majority of people live in polluted areas. With the improvement of evaluation criteria, economic damage of respiratory disease caused by PM_10_ is much bigger. The health effects of Lanzhou urban residents should not be ignored. The government needs to find a better way to balance the health of residents and economy development. And balance the pros and cons before making a final policy.

## Background

The emission of harmful atmospheric pollutants, such as nitrogen oxides, sulfur oxides, soot, dust, smoke and other suspended particulate matter, can harm human health. The World Health Organization (WHO) has found that there are more than 2.7 million deaths worldwide attributable to air pollution each year [1]. Air pollution has become one of the most visible environmental problems in China, because of massive coal combustion with inadequate emission controls (Wang and [2] and motor vehicle emissions. Also, natural sources such as sandstorms are important, especially in North China, such as the area around Lanzhou [3-6].

In Europe and in the United States, suspended PM is recognized as the most important air pollutant in terms of human health effects [7]. Air pollution levels in developed countries have decreased dramatically in recent decades. In 2004, a study was done to assess the health impacts of by PM_10_ pollution (less than 10 microns in diameter) in 111 key Chinese cities. None of these cities have attained the national first class ambient air quality standard, and over half currently exceed the national second class standard, The total economic cost caused by PM10 pollution was estimated as approximately USD 29,178.7 million [8]. Serious PM_10_ levels are prevalent in many cities of China. PM is the most important air pollutant in the northern part of the country [9].

Numerous epidemiological studies indicate that both long- and short-term exposure to atmospheric PM, especially PM_10_, are associated with increases in mortality and morbidity [10-14]. Young children, the elderly, individuals with predisposed diseases, such as cardiovascular and pulmonary diseases, and workers in certain industries may be at higher risk. This is because of their increased biological sensitivities and different exposure patterns [15,16]. The relative risks of PM10 were 1.045 for population with the age less than 15-year-old, 1.033 for population with the age more than 65-year-old, 1.023 for male, 0.990 for female and 1.011 for population with the age between 15-year-old and 65-year-old [17].

Association with air pollution has been studied for a range of diseases, such as asthma, lung irritation, bronchitis, pneumonia, premature death and heart disease [15-22]. Zanobetti et al. [23] examined the association between PM_10_ and hospital admissions for heart and lung disease in 10 cities in the United States. They found a 2.5% (95% confidence interval (CI): 1.8–3.3%) increase in chronic obstructive pulmonary disease, a 1.95% (95% CI: 1.5–2.4%) increase in pneumonia, and a 1.27% increase (95% CI: 1–1.5%) in cardiovascular disease for a 10 μg m^-3^ increase in PM_10_. Wellenius et al. [24] analyzed the association between PM_10_ and congestive heart failure in seven US cities. The APHEA 2 (Air Pollution on Health: European Approach) project investigated short-term health effects of particles in eight European cities. This study confirmed that particle concentrations in these cities were positively associated with increased numbers of admissions for respiratory diseases [25]. The evidence of adverse health effects related to PM_10_ is consistent with various cities in China. This is even more important in China because of its severe PM_10_ pollution levels and associated high population densities. Many studies in Chinese cities have shown health effects on mortality and morbidity associated with exposure to PM similar to studies in Europe and America. The “Impact of Air pollution on Children’s Lung Function” study is a Sino–US scientific and technological collaboration. Three size fractions of PM (PM_2.5_, PM_2.5–10_, and PM_10_) were measured in schoolyards at eight elementary schools in four large Chinese cities (Guangzhou, Wuhan, Lanzhou, Chongqing) during 1995 and 1996. The results gave significant evidence that there were positive associations between outdoor PM levels and pediatric respiratory symptoms [26]. Using factor analysis with varimax rotation, sources of fine and coarse airborne PM in the four cities were examined. The contribution to coarse PM (PM_2.5–10_) from crustal factors is greater than that to fine PM (PM_2.5_) [27].

Relevant research has been done in Chinese cities such as Shanghai [28], Beijing [29-31], Wuhan [32] and Hong Kong [33]. Lanzhou is expanding and there is an influx of population from the rural areas and urban fringe to the core of the city. Air quality in the city has been deteriorating, resulting in one of the most serious pollution problems nationally [34-36]. For Lanzhou, quantitative evaluation of the adverse health effects of PM_10_ pollution is becoming critical to optimizing the energy structure, controlling total emission of air pollutants, and the treatment of automobile exhaust. Referencing to the related epidemiological literature published around the world in recent years, Hou et al. screens out the PM_10_ health endpoints and appropriate exposure-response coefficients, and calculates the health economic loss by PM_10_ of Lanzhou during 2002–2009. But there is a hypothesis the uniform distribution of PM_10_. In fact, it is the uneven distribution, so the health economic loss by PM_10_ of Lanzhou is calculated under this scenario.

The present work, based on ESRI ArcGIS Server 9.2 (Geographic information system) spatial information analysis function, assesses the harmful health effects of PM_10_ pollution in Lanzhou using monitoring and population data. To provide a more detailed picture of exposure, population exposure levels are estimated by combining PM_10_ spatial distribution with that of population. We selected the generalized additive model (GAM), run with R software, to determine health effects of PM_10_ on morbidity rates and to establish an exposure-response function for PM_10_. Economic costs of adverse health effects related to PM_10_ are also calculated. The results can serve as a useful reference for health risk management, and as a scientific basis for reducing economic loss and policy making regarding air pollution control.

## Methods

### PM_10_ data collection

This case study is for Lanzhou, the capital of Gansu Province and an industrial and oil processing city in northwest China. Lanzhou is surrounded by high mountains; the frequency of calm winds is as much as 75%, and 87% in winter. Temperature inversions occur year round. Such inversions, calm winds, and a continuously stratified atmosphere are not conducive to horizontal and turbulent diffusion within basins [37].

**Figure 1:**
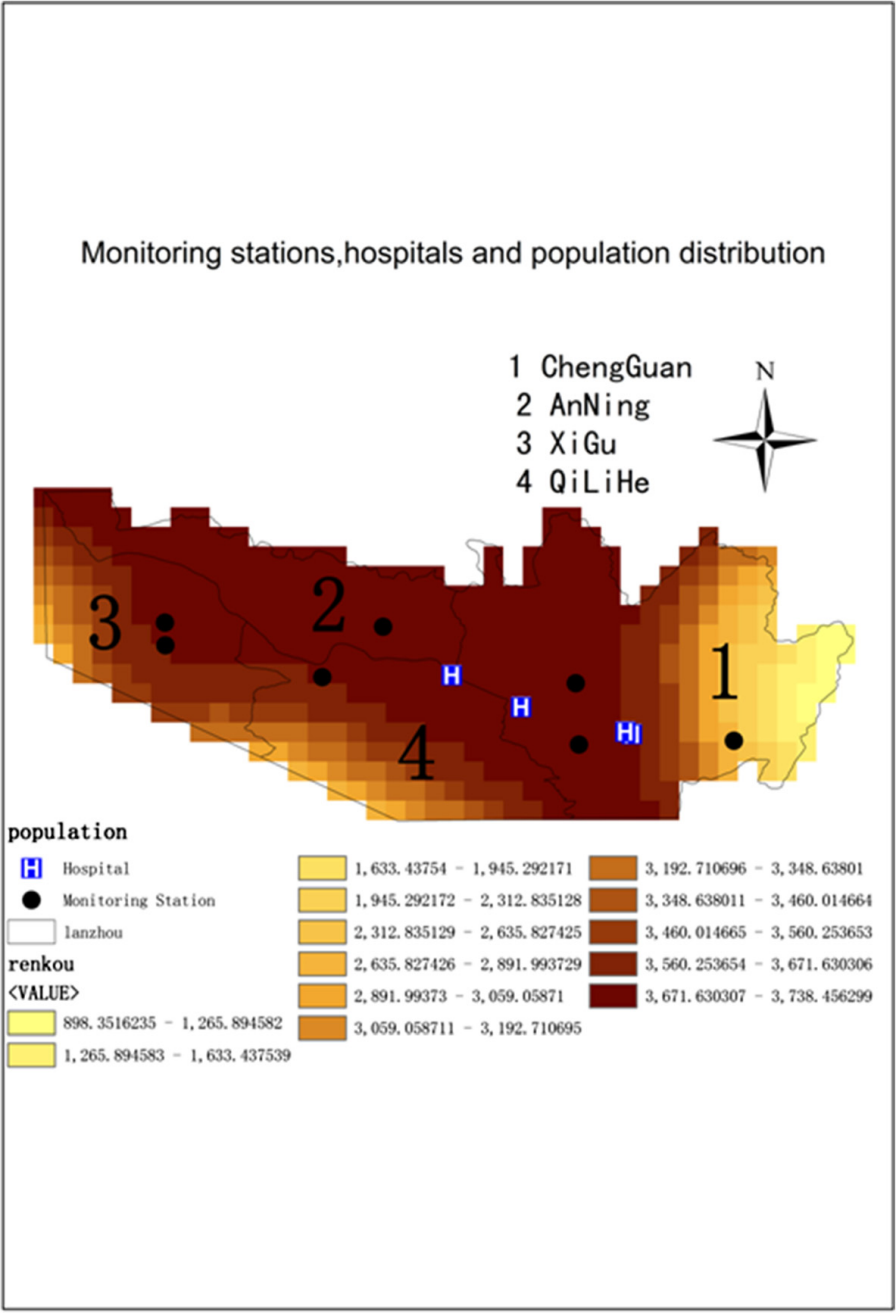
**Total suspended particles (TSP) monitoring stations, four hospitals and population distribution in the Lanzhou urban areas during 2000 (The units are number of people per km**^**2) **^**in Lanzhou urban areas (China) during 2000.**

Total suspended particulate (TSP) concentration data from monitoring stations were taken from the Project of “Science and Technology Cooperation Project of the Chinese Academy of Science and Gansu Province”. Seven ambient air quality monitoring stations were distributed relatively across the Lanzhou urban area, including the XiGu, ChengGuan, AnNing, and QiLiHe districts. All stations made measurements in 2000. Because the Project did not include direct measurements of PM_10_ concentrations, a conversion factor between TSP and PM_10_ was used. The distribution of the seven stations and four hospitals is shown in Figure 1. The conversion factor chosen is similar to that proposed by Dockery et al. [38]. The PM_10_ concentration is calculated as

(1) $$PM_{10}=\mathrm{TSP}\times 0.55$$

The PM_10_ concentration data were used for calculation of daily exposure and population-weighted concentration.

### Population data for Lanzhou

Data of the Gridded Population of the World version 3 (GPWv3) from the Center for International Earth Science Information Network (CIESIN) of Columbia University and Centro Internacional de Agricultura Tropical (CIAT) were used as basic population data. GPWv3 is the latest update of the GPW dataset. Earlier versions and GPWv3 have been extensively used in global population studies [39-41].

### Data for fitting of GAM

We obtained daily SO_2_, NO_2_ and PM_10_ concentration data from the Lanzhou Environmental Monitoring Station. These daily data cover the 5 years from 2001 to 2005. The meteorological data are daily maximum, minimum and average temperature, daily atmospheric pressure and relative humidity, and were provided by the Lanzhou Meteorological Bureau. These data are also from 2001 to 2005. Hospital admissions data of respiratory system diseases are from the four largest comprehensive hospitals in the Lanzhou urban area.

### Morbidity data

Hospital admissions data of respiratory system diseases are considered as health endpoints. Lacking access to data of morbidity rate of respiratory system diseases in Lanzhou, this rate is calculated in accord with ratios of national hospital admissions for respiratory system diseases from the Chinese Ministry of Health. Methods International classification (ICD-10) was adopted to provide the scientific basis for statistical analysis to calculating the disease composition. Specific classification standard is ICD-10:J00-99. Diseases of the information collected are from each hospital database.

### Calculation of daily exposure and population-weighted concentration

Based on the GIS, PM_10_ concentration data from the seven monitoring stations were spatially interpolated, using kriging spatial interpolation. PM_10_ concentrations were calculated at resolution 0.01° × 0.01°, approximately 1 km × 1 km. Population data were also spatially interpolated, to attain the high spatial resolution (0.01° × 0.01°) matching that of PM_10_ concentration data. Using the GIS spatial information analysis function, the population and PM_10_ concentration layers (total of 475 grid points) were overlaid to analyze population exposure levels in various concentration ranges.

Figure 1 shows that the four districts of the Lanzhou urban area are all densely populated. Population of the ChengGuan district is more concentrated, followed by AnNing, QiLiHe district and XiGu districts, which are all distributed in a narrow, long belt. There are 43 grids, over which population density ranged from 800 km^-2^ to 2700 km^-2^ mainly in the eastern ChengGuan district. Average population density is greater than 3700 km^-2^ mainly in the AnNing and western ChengGuan districts with 213 grids, comprising 45% of the total grid. In the XiGu and QiLiHe districts, spatial gradients of population density are relatively large, and the direction is northwest-southeast. The gradient in the ChengGuan district is east–west.

We also calculated the population-weighted exposure level (PWEL). Given grid i, the population weighted exposure equation is as follows:

(2) $$\text{PWEL}\;=\;{\sum \left(P_{i}\;\times \;C_{i}\right)/\sum P}_{i},$$

where P_i_ is the population in grid i, and C_i_ is its average PM_10_ concentration.

**Figure 2:**
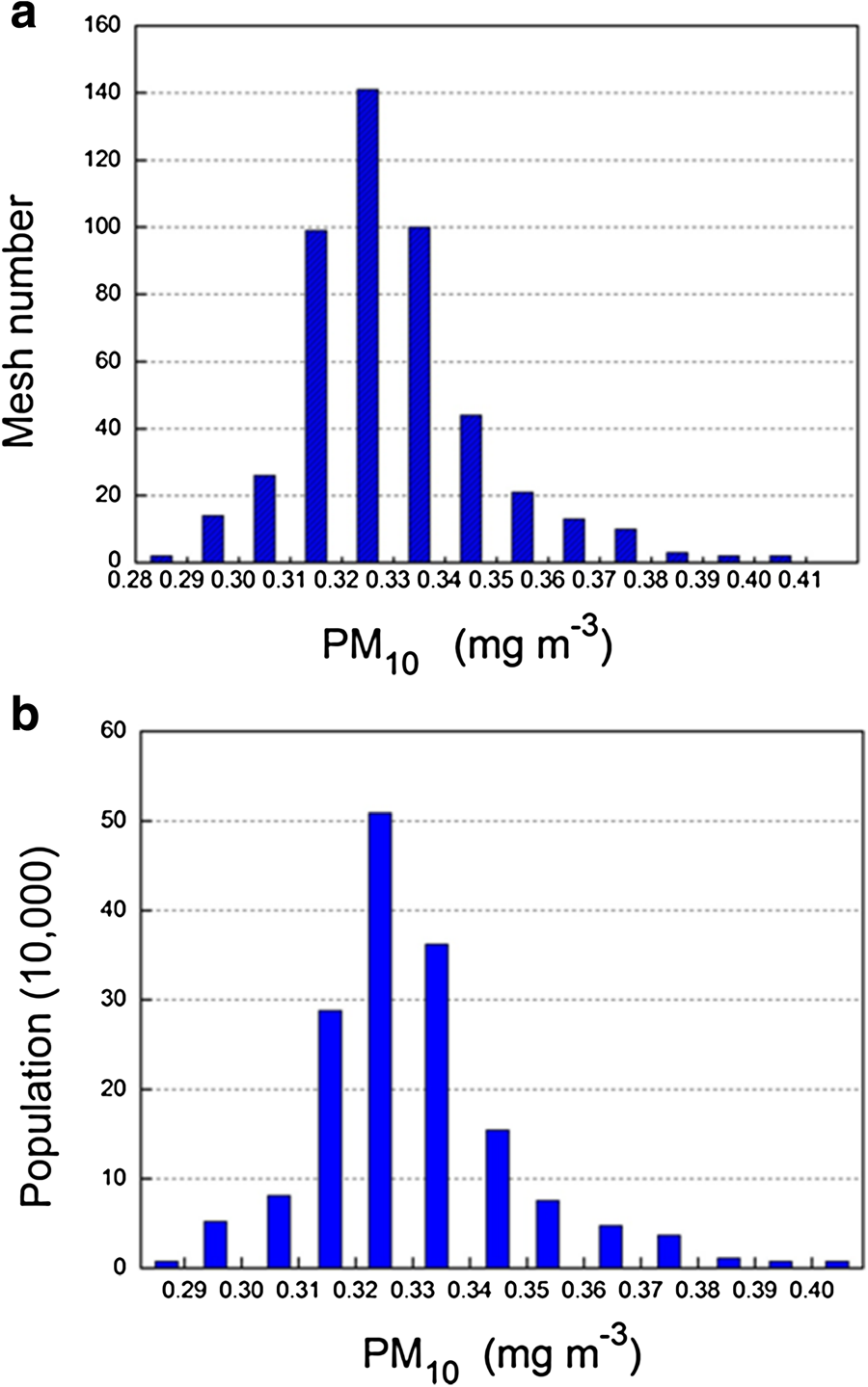
**Population exposure level in Lanzhou urban areas for PM**_**10 **_**(a) Mesh number in different PM**_**10 **_**concentration intervals.(b)** Frequency distribution of population weighted exposure concentrations.

Figure 2(a) reveals that PM_10_ concentrations in most Lanzhou urban areas are between 0.31 and 0.35 mg m^-3^ before weighting. Therefore, the population within grids with PM_10_ concentration between 0.31 and 0.35 mg m^-3^ has the greatest exposure. After population weighting (Figure 2(b)), we see the following results. There are 140,000 people exposed to between 0.28 mg m^-3^ and 0.31 mg m^-3^, more than 150,000 exposed to between 0.31 mg m^-3^ and 0.34 mg m^-3^, more than 270,000 exposed to between 0.34 mg m^-3^ and 0.37 mg m^-3^, and more than 60,000 people exposed to between 0.37 mg m^-3^ and 0.41 mg m^-3^. The grid average of PM_10_ concentration in all areas is 0.329 mg m^-3^, and the population-weighted average is 0.345 mg m^-3^, an increase of nearly 5%. With population weighting, there are more people living in more polluted areas. Consequently, it is more realistic to use the population-weighting algorithm for determining population exposure to PM_10_ concentration.

### Fitting of GAM

The GAM was originally described by Hastie and Tibshirani [42,43], and has been applied in a variety of research fields. In time series studies, the GAM was used to adjust for potential confounders of seasonality, trends, and weather variables in epidemiological analyses of mortality. Here, the *mgcv* package with R2.6 [44] was used to assess the relationship between the daily PM_10_ concentration and daily hospital admissions for respiratory diseases. GAM was set up based on the above package, which is largely based on Hastie [42,43]. This package has an advantage in terms of computational time, and is used when the database is large.

The Akaike information criterion (AIC) was proposed by Akaike in 1973. A smaller AIC is characteristic of a model with better fit. We selected the best form of the model by minimizing AIC [42], which is achieved by adjustment of the degrees of freedom (*df*).

The GAM fitted to the respiratory admission time series Y_k_, under the assumption of Poisson counts and a log-link function for predetermined lags is

(3) $$\begin{array}{l} \text{Log}\;\left[E\;\left(Y_{k}\right)\right]\;=\alpha +\text{DOW}+\beta \;PM_{10}\\ \;+s\;\left(\text{time},\;\text{df}\right)\;+s\;\left(Z_{k},\;\text{df}\right) \end{array}$$

In this equation, α denotes some constant, β the exposure-response coefficient of PM_10_, and s ( ) the smoothing spline function for optimum flexibility when modeling the confounders. DOW is a dummy variable of weekday effect, and time is calendar time. *df* is an adjustable variable according to various results for the AIC.

Maximum, minimum and average temperature, daily atmospheric pressure and relative humidity are considered confounders. DOW, time, maximum temperature, daily atmospheric pressure and PM_10_ concentration are involved in the GAM, toward obtaining the AIC. The results are significant when adding other factors makes no difference in fitting the GAM. When the AIC value calculated by different models with various factors can no longer be diminished, the result is the final model.

### Exposure-response function

The exposure-response function is often used in epidemiological studies to relate air pollution and adverse health outcomes. For the exposure population, disease or death is a small-probability event following a Poisson distribution.

(4) $$E‒E_{0}\;\text{exp}\;\left[\beta \;\left(C-C_{0}\right)\right]$$

Morbidity and excess mortality caused by a certain type of pollutant is calculated as

(5) $$N=\;P\;\left(E‒E_{0}\right)=PE\;\left(1-1/\exp\left[\beta \;\left(C-C_{0}\right)\right]\right)$$

In Equations (4) and (5), β (per 1 μg m^-3^) is the exposure-response coefficient; C is PM_10_ concentration (μg m^-3^); C_0_ is threshold concentration (μg m^-3^); E (%) and E_0_ (%) are corresponding health effects C and C_0_; P (persons) is exposure population; and N (persons) is morbidity or excess mortality numbers caused by a certain type of pollutant. E can be derived if data are available for β, C, C_0_, and E_0_. The exposure-response function is a quantitative functional relation between the variation of PM_10_ and health endpoint. The difficulty of establishing this function is choosing β and C_0_. Here, β is calculated with the GAM. It was shown in an Australian study [45] that although levels of particulate air pollution in Sydney were low, PM pollution was consistently associated with both daily mortality and hospital admissions. There was no deterministic threshold concentration for health effects presented. In this work, we chose three threshold concentrations to evaluate economic costs of the adverse health effects of PM_10_ pollution. The WHO report “Air Quality Guidelines Global Update 2005” has indicated that no matter how low PM concentration was, it could harm human health [46]. As a result, zero (mg m^-3^) was chosen as the PM_10_ threshold concentration, used to calculate “no harmful health effects.” We also used the second threshold concentration recommended by the WHO, 20 μg m^-3^. The national first class standard in China, 50 μg m^-3^, was used as the third. Because C_0_ is a very important and sensitive parameter in estimating the health effect of pollutants, the purpose of selecting different ones was to ensure evaluation objectivity. Economic losses were determined for C_0_ at 0 μg m^-3^, 20 μg m^-3^ and 50 μg m^-3^.

### Determining economic costs of adverse health effects

Once the relationship between PM_10_ concentration and health effects is established, the next stage requires assessment of economic costs versus those predicted based on health effects. Based on the GIS, average daily cases of respiratory disease caused by PM_10_ on the 0.01° × 0.01° grid can be calculated according to β (exposure-response coefficient) and actual incidence rate of the disease. Total cases of the disease are obtained by summing daily cases over an entire year.

There are no results for cost of pollution-related health effects for inpatients and outpatients in China using the willingness to pay approach, so the cost of illness (COI) is used to estimate economic costs of adverse health effects of PM_10_ pollution.

(6) P=fp=ft

(7) $$P=\mathrm{fp}\;\left(1+△C\;\beta \right)\;$$

(8) $$P=\mathrm{fp}\;\left(△C\;\beta /\left(1+△C\;\beta \right)\right)$$

P is super hospital visitors under the present air pollution levels; fp is hospital visitors, under the present air pollution levels from China health statistics yearbook; ft is clean thick degree level in the hospital; β is the exposure-response coefficient; △C is the difference between the health hazard pollutants concentration threshold and actual pollutant concentration.

## Results

Based on the GIS spatial information analysis function, health effects of PM_10_ in Lanzhou were evaluated by use of monitoring and population data. Population data were spatially interpolated to attain the higher spatial resolution matching that of PM_10_ concentration data. The results indicate that air in Lanzhou is seriously polluted by PM_10_ in winter and spring, which would greatly impair human health and produce more health-related economic costs. In evaluating these costs, results varied with the calculation scenario.

The 95% confidence limit of β is [β ± 1.96SE (standard error SE)] and PM_10_ lag 4 is significantly related to respiratory system disease (β = 0.197; SE = 0.061; 95% confidence limit 0.317–0.077). Because dust is the main component of PM_10_ in Lanzhou, toxicity is relatively low. Although PM_10_ concentration is high, the exposure response relation coefficient (RR) is relatively small, as shown in Table 1. The smallest RR is 1.009(1.006-1.013) and biggest is 1.020(1.015-1.024). RR for a 10 μg m^-3^ increase of PM_10_ in Lanzhou is between 1.002 and 1.003.

**Table 1:** **Comparison of RR for a 10 μg m**^**-3**^**increase of PM10 in different areas**

**Case**	**RR**	**95% CI**
Tacoma(Schwartz,1995)	1.019	1.006~1.032
Spokane(Schwartz,1996)	1.016	1.007~1.026
London(Atkinson,1999)	1.010	1.004~1.016
APHEA(Atkinson,2001)	1.009	1.006~1.013
USA(Zanobetti,2000)	1.020	1.015~1.024
Ontario(Schwartz,1995)	1.012	1.008~1.016
New York(Schwartz,1995)	1.010	1.002~1.019
New Haven(Schwartz,1995)	1.012	1.000~1.025

Cases in different scenarios for C_0_ were calculated using Equation (5), and the 95% confidence limit is given in Table 2. The results show that the excess number of patients decreased with C_0_ increase. This indicates that the excess number of patients is sensitive to the selection of C_0_, as are total costs. When the threshold concentration is relatively high, the proportion of economic costs for PM_10_ to the GDP is large.

**Table 2:** **Economic Evaluation in economic cost of health harming effecting by PM**_**10**_**pollution under different scenarios of C**_**0**_

***Scenario***	***Standard***	***C0***	***Cases***	***Cost***	***Total cost (﹩)***	***GDP***	***GDP***
		***(μg m***^***-3***^***)***		***(per-case) (﹩)***		***(﹩ in billions)***	***Percentage (%)***
A	zero	0	9651.9 (15230.5, 3847.6)	660	6370254 (10052130, 2539416)	3.096	0.206
B	WHO	20	9083.9 (14350.9, 3616.9)	660	5995440 (9471594, 2387154)	3.096	0.194
C	National first-class standard	50	8227.8 (13021.1, 3270.2)	660	5430348 (8593926, 2158332)	3.096	0.175

We calculated coefficients of correlation between the seven monitoring stations. The PM_10_ time series of Wen Hua Gong station was used to investigate temporal variation. Its correlation coefficient with other stations was as high as 0.83 (above 99% confidence level), indicating satisfactory representation of the Lanzhou urban area.

**Figure 3:**
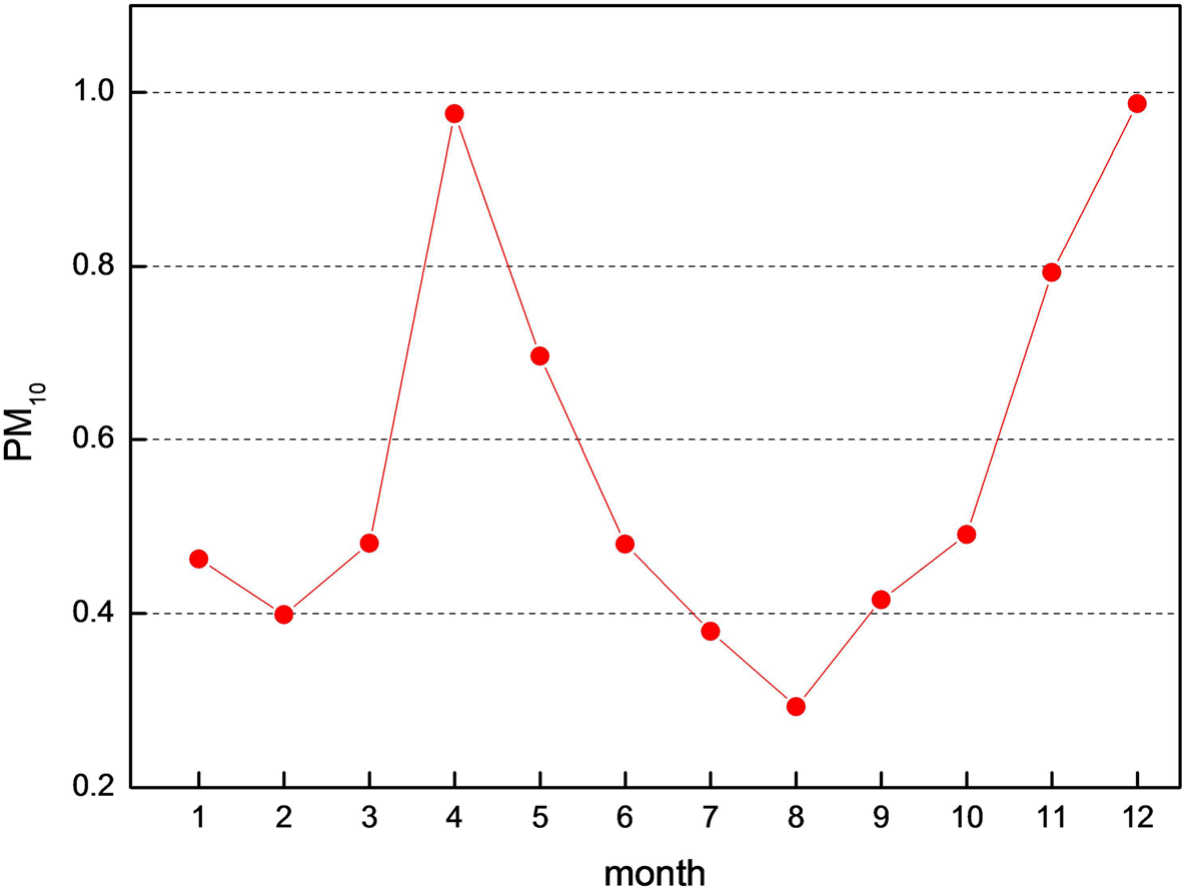
**PM**_**10 **_**concentration variation at the Wen Hua Gong station during 2000 (Unit:mg/m**^**3**^**).**

Lanzhou is within the Hexi Corridor, in an inland arid or semi-arid area of China. It is one of the most polluted cities in the country. Figure 3 shows that PM_10_ concentration in Lanzhou peaks in April and December. Sandstorms frequently impact Hexi Corridor areas, and represent the greatest moving source of pollution for Lanzhou. In spring 2000, when precipitation was low and windy weather was caused by frequent activities of cold air, the topsoil was dry and could be readily blown away. These were all dynamic factors for sandstorm formation. Therefore, the peak in April was mainly caused by sandstorm-related weather; after spring, the PM_10_ concentration fell sharply. The minimum concentration was in August. The contribution of coal combustion for domestic heating to winter PM_10_ pollution was very marked, and released large amounts of harmful pollutant. Inversion layers near the ground and basin landforms were not conducive to horizontal and turbulent diffusion, and constituted the major reason for the peak in December.

Because Lanzhou is at the northern periphery of East Asian summer monsoon activity, precipitation is very irregular. In summer 2000, the East Asia trough strengthened and moved west, causing cold air to move southward and collide with warm air from the southern airstream of the subtropical high, generating strong winds and extensive precipitation in Lanzhou. Therefore, the PM_10_ concentration was relatively low in June, July and August.

With consideration of available research findings and data, we chose COI as an economic evaluation method [47]. Given the sensitivity to C_0_, we used three different levels for it to ensure evaluation objectivity.

For C_0_ of 0 μg m^-3^, 20 μg m^-3^ and 50 μg m^-3^, the proportions of economic cost to GDP were 0.206%, 0.194% and 0.175% respectively. Thus, PM_10_ abatement is necessary. To reduce PM_10_ concentration in Lanzhou, cutting atmospheric pollutant emissions, planting surrounding mountains with greenery, and developing sandstorm monitoring and early-warning technology are all effective approaches.

## Discussion

C_0_ is a sensitive parameter in estimating the health effect of pollutants, when the threshold concentration chosen relatively high, the economic costs of adverse health effects of PM_10_ pollution is low, when the selected threshold concentration is relatively low, the high economics cost of adverse health effects of PM_10_ pollution.

In establishing the exposure-response function, we used the GAM with R software to quantify the adverse health effects of PM_10_. Different models can produce varying estimates. We selected the GAM because it is more flexible, and allows nonparametric adjustments for nonlinear confounding affects of seasonality, the weekday effect and weather variables. Moreover, the R software was more computationally steady than S-Plus software.

We found that although PM_10_ concentrations are much higher in the Lanzhou urban area and the urban population their experiences much greater exposure than in other Chinese cities [36,48,49], the exposure-response coefficient β is relatively small, suggesting weak sensitivity of the population in local areas. The population structure of Lanzhou is different from that of Europe and the United States where there is a larger aging population; the elderly are an easily affected group. Another reason may be that PM_10_ components are different from other areas. In developed countries and areas, the main particle pollution source is automobile exhaust, which generates secondary particles that are more toxic than the PM_10_ of Lanzhou. Urban dust and sand have high proportions of local PM_10_ in the city, which are also less toxic.

The relationship of TSP and PM_10_ is quite different related to the source and it can be described in different equation. The constant conversion factor between TSP and PM_10_ due to the lack of direct PM_10_ measurement, the dismatch between the 2000 population data and the time period of hospital admissions (2001 to 2005), limited monitors(7 sites), and respiratory hospital admission from only four hospitals. it can also lead to uncertainty.

Current time series research on adverse health effects of air pollution in China is focused on “one point,” i.e., it considers research areas as a single point. In fact, the relationship between PM_10_ concentration and population distribution can change over time and space. In this study, there were seven monitoring stations in the research area, and PM_10_ concentration data were interpolated by the GIS. We evaluated health effects related to PM_10_ and their economic costs. We transformed fixed-site pollution monitoring observation to a continuous surface. This is a predictive technique for PM_10_ concentrations in areas without observed data, causing inevitable data deviation. More monitoring sites and atmospheric pollution models for simulating pollutant concentration are useful means to decrease such deviation. Choi et al. [50] showed that health effects in some areas were more significant upon combining PM monitoring concentration data and Models-3 CMAQ (Community Multiscale Air Quality Modeling) simulated results for PM.

## Conclusions

The average population-weighted PM_10_ concentration is 0.345 mg m^-3^ more than that without population weighting, an increase of nearly 5% from 0.329 mg m^-3^ to 0.345 mg m^-3^. Considering population spatial distribution in Lanzhou, there are larger population living in more polluted areas.

We select threshold concentration C_0_ at: 0 μg m^-3^ (no harmful health effects), 20 μg m^-3^ (recommended by the World Health Organization), and 50 μg m^-3^ (national first class standard in China) to calculate excess morbidity cases. For these three scenarios, proportions of the economic cost of PM_10_ pollution-related adverse health effects relative to GDP are 0.206%, 0.194% and 0.175%, respectively.

## Competing interests

The authors declare that they have no competing interests.

## Authors’ contributions

ZS carried out the calculation of GAM model and participated in drafting the manuscript. XA participated in the design of the study and drafting the manuscript. YT participated in the GAM model calculation and the statistical analysis. QH participated in the calculation of health-related economic costs. All authors read and approved the final manuscript.

## Pre-publication history

The pre-publication history for this paper can be accessed here:

http://www.biomedcentral.com/1471-2458/13/891/prepub
